# Development of a Rapid, Sensitive, and Visual Recombinase-Aided Amplification Combined with *Pyrococcus furiosus* Argonaute (RAA-*Pf*Ago) Assay for Sex Identification in Swinhoe’s Pheasant (*Lophura swinhoii*)

**DOI:** 10.3390/cimb48101002

**Published:** 2026-09-29

**Authors:** Tongtong Zhan, Fanrong Liu, Huan Zhang, Shenluan Tan, Xuanjiao Chen, Tanzipeng Chen, Lu Liu, Guoqian Wang, Jiaqi Sa, Shouquan Zhang, Hengxi Wei, Li Li, Fanwen Zeng

**Affiliations:** 1 State Key Laboratory of Swine and Poultry Breeding Industry, College of Animal Science, South China Agricultural University, Guangzhou 510642, China; 2 Beijing Key Laboratory of Captive Wildlife Technologies, Beijing Zoo, Beijing 100081, China; 3 Guangzhou Zoo & Guangzhou Wildlife Research Center, Guangzhou 510075, China

**Keywords:** Swinhoe’s pheasant, sex identification, recombinase-aided amplification (RAA), *Pyrococcus furiosus* Argonaute (*Pf*Ago)

## Abstract

Swinhoe’s pheasant (*Lophura swinhoii*) exhibits pronounced sexual dimorphism in adults. However, sexual differences are minimal in juveniles and subadults, making accurate early sex determination essential for captive breeding and conservation. Conventional PCR-based sexing methods require expensive equipment and are unsuitable for field or basic breeding facilities. We developed a novel recombinase-aided amplification (RAA) combined with a *Pyrococcus furiosus* Argonaute (*Pf*Ago) nuclease system targeting the W-linked EE0.6 sequence (EE0.6–RAA-*Pf*Ago) for rapid and visual sex identification in Swinhoe’s pheasant using DNA extracted from feather samples. In addition, a UCE-based RAA-*Pf*Ago internal amplification control (UCE–RAA-*Pf*Ago) was established to monitor DNA quality and amplification success. The optimal EE0.6-RAA reaction was performed at 39 °C for 20 min, followed by *Pf*Ago-mediated product detection. The entire assay can be completed within 50 min, and results are visually interpretable under blue light. In our dilution experiments, the EE0.6–RAA-*Pf*Ago assay showed a detection limit of 0.65 ng of genomic DNA, which was 10-fold lower than that of conventional PCR (6.5 ng). In a blind test of 19 field feather samples, the results were 100% concordant with conventional PCR. This study establishes a rapid, sensitive, convenient, and equipment-minimizing method for sex identification in Swinhoe’s pheasant, suitable for field applications and basic captive breeding facilities.

## 1. Introduction

Swinhoe’s Pheasant (*Lophura swinhoii*), also known as the Blue Pheasant, is a member of the genus *Lophura* (family Phasianidae) and is endemic to Taiwan Island [1]. It is one of the most endangered pheasant species and is classified as Vulnerable on the IUCN Red List and as a First-Class National Key Protected Wild Animal in China [2]. Due to extensive habitat destruction caused by historical deforestation, agricultural expansion, and infrastructure development in mid- to low-elevation primary and secondary broadleaf forests, combined with past overhunting and ongoing human disturbances, the population of this species has declined sharply [3,4,5]. Conservation measures, including habitat protection and restoration, legal protection, population monitoring, and captive breeding, are urgently needed. Captive breeding followed by reintroduction is a key strategy for the conservation of this endangered species [6,7]. In captive breeding programs, the inability to accurately determine the sex of juveniles and subadults has been identified as a significant management bottleneck, as it can lead to suboptimal pairing, skewed sex ratios, and reduced reproductive output [8,9]. For instance, improper sex ratios in reintroduction programs can compromise genetic diversity and limit population growth [7]. Accurate sex identification, especially in juveniles and subadults, is critical for optimizing breeding pairs, maintaining a balanced sex ratio, and enhancing reproductive output in ex situ conservation programs. Although adult Swinhoe’s pheasants exhibit conspicuous sexual dimorphism, sex-related differences in juveniles and subadults are extremely subtle [10], rendering conventional morphological methods—such as plumage assessment, body morphology, and cloacal sexing—unreliable [11]. Therefore, a rapid, sensitive, and field-deployable molecular method for sex identification is urgently needed for effective conservation and scientific management.

In birds, sex is genetically determined by the ZW system, where males possess two Z chromosomes (ZZ) and females carry one Z and one W chromosome (ZW). Most birds can be molecularly sexed by PCR-based detection of sex-linked differences, such as the intronic length polymorphism of the chromodomain helicase DNA-binding protein 1 (CHD1) gene between the Z and W chromosomes or the presence of the W-specific 0.6-kb EcoRI sequence (EE0.6), a non-repetitive EE0.6 sequence conserved on the avian W chromosome [12,13]. However, conventional PCR-based sexing typically targets CHD1, but it requires expensive thermal cyclers, takes 2–3 h, depends on post-amplification gel electrophoresis, and needs relatively high DNA input, limiting its use in field settings or basic breeding facilities [14,15,16]. Therefore, a rapid, sensitive, and field-deployable molecular method is urgently needed.

To overcome these logistical barriers, isothermal nucleic acid amplification techniques have been developed as promising alternatives. Loop-mediated isothermal amplification (LAMP) has been adapted for avian sexing in species such as the red-whiskered bulbul, offering rapid results, but its primer design is notoriously complex (requiring six primers) and it is prone to non-specific amplification [15]. Recombinase-aided amplification (RAA) and its close relative recombinase polymerase amplification (RPA) circumvent these issues by employing a simple two-primer system and operating at a constant low temperature (37–42 °C) with fast reaction kinetics (typically 10–30 min) [17]. Despite these advantages, current RAA/RPA-based sexing assays still rely on lateral flow strips or real-time fluorescence readers for result interpretation, which adds cost and operational complexity.

To address the remaining gap for a method that is field-deployable after a simple DNA extraction step and requires minimal equipment, we introduced the *Pyrococcus furiosus* Argonaute (*Pf*Ago) nuclease into the detection system. Unlike CRISPR/Cas systems that require protospacer adjacent motifs (PAMs) for target recognition and suffer from off-target collateral effects [18], *Pf*Ago is PAM-free and employs short single-stranded DNA guides for specific cleavage [19]. Furthermore, the 5′-phosphorylated cleavage products of *Pf*Ago can act as new guide molecules to initiate cascade cleavage, enabling signal amplification without compromising specificity [20]. Previously, we have successfully applied the RAA-*Pf*Ago platform to determine the sexing in other avian species, such as flamingos (*Phoenicopteridae*), demonstrating its broader applicability and robustness across different taxa [21]. To further ensure reliable diagnostic performance, we incorporated a UCE-based internal amplification control (IAC), which is essential to distinguish true negatives from false negatives caused by DNA degradation, insufficient template, or reaction inhibition [22]. To our knowledge, no molecular sexing method has been reported for Swinhoe’s pheasant. Therefore, in this study, we developed a rapid and visual RAA-*Pf*Ago assay targeting the W-linked EE0.6 sequence. Unlike CHD1, EE0.6 is absent from the Z chromosome, so it produces a female-specific presence/absence signal. Building upon these established features, the specific innovations and contributions of the present study are threefold. First, we extend the RAA-*Pf*Ago platform to a novel avian species (*Swinhoe’s pheasant*), demonstrating its adaptability to a new sex-linked target locus (W-linked EE0.6 sequence) with distinct sequence characteristics. Second, we demonstrate that the PAM-free nature of *Pf*Ago is particularly advantageous for this specific application, as our EE0.6 amplicon lacks convenient PAM sites within the optimal primer-binding regions that would have precluded CRISPR/Cas-based detection. Third, we established a UCE-IAC that amplifies in both sexes, enabling reliable discrimination between true negatives and false negatives in field samples. This study combined RAA with *Pf*Ago to establish a rapid, sensitive, convenient, and visually interpretable detection system targeting the female-specific W-linked EE0.6 sequence gene, thereby providing a practical tool for the conservation and management of the endangered Swinhoe’s pheasant.

## 2. Materials and Methods

### 2.1. Sample Collection

Naturally molted feather samples were collected from 25 Swinhoe’s pheasants in a captive breeding population at Guangzhou Zoo in Guangdong Province, China. This captive population was established from Taiwan-endemic individuals and is maintained for conservation and breeding purposes. Among the 25 samples, 6 individuals with known sex (3 females and 3 males) were used for specificity evaluation, 19 individuals of unknown sex (juvenile/subadult birds) were used for field validation. The sex of the 6 known-sex individuals was independently determined by conventional CHD1 PCR using primers 2550F/2718R (Sangon Biotech Co., Ltd., Shanghai, China) and, where possible, by adult morphology [23]. The 19 clinical samples were from juvenile/subadult birds whose sex was initially unknown. Each sample was individually coded and stored in a coded plastic bag and frozen at −20 °C until use. To prevent cross-contamination, all feather samples were handled with sterile forceps, and each sample was stored in an individually sealed plastic bag. DNA extraction was performed in a dedicated pre-amplification area physically separated from post-amplification handling. Negative controls (ddH_2_O) were included in all PCR and RAA-*Pf*Ago assays to monitor for contamination. All animal experimental protocols were approved by the Animal Use and Care Committee of Guangzhou Zoo and conducted in accordance with the ARRIVE guidelines.

### 2.2. DNA Extraction

The basal 2–5 mm portion of the feather shaft (calamus), which contains the most cellular material, was excised and placed in 1.5-mL Eppendorf tubes. Total genomic DNA was extracted using the HiPure Tissue and Blood DNA Kit (D3018; Megan, Guangzhou, China). DNA concentration and purity were measured using a NanoDrop ND-2000 spectrophotometer (ThermoFisher Scientific, Waltham, MA, USA). The A260/A280 ratios ranged from 1.8 to 2.0, and the A260/A230 ratios ranged from 2.0 to 2.2, indicating acceptable DNA purity and no significant inhibitor contamination. DNA was stored at −20 °C until use.

### 2.3. Conventional PCR Assay

Conventional PCR was performed to amplify the CHD1 gene as a reference sexing method using primers 2550F/2718R, which were originally described by Fridolfsson and Ellegren [23]. The 20-μL reaction mixture containing 2 μL of DNA template, 10 μL of 2× DreamTaq PCR Master Mix (K1081; ThermoFisher Scientific), 6 μL of ddH_2_O, and 1 μL each of forward and reverse primers (10 μM): 2550F (5′-GTTACTGATTCGTCTACGAGA-3′) and 2718R (5′-ATTGAAATGATCCAGTGCTTG-3′). The thermal cycling protocol was: initial denaturation at 94 °C for 3 min; 35 cycles of 94 °C for 30 s, 49 °C for 45 s, and 72 °C for 1 min; and a final extension at 72 °C for 5 min. Products were separated by 1.5% agarose gel electrophoresis and visualized under UV light (Bio-Rad, Hercules, CA, USA). CHD1-Z yielded a band of approximately 600 bp, while CHD1-W yielded an additional band of approximately 450 bp. Therefore, females (ZW) showed two bands (approximately 600 and 450 bp), whereas males (ZZ) showed a single band (approximately 600 bp). For conventional PCR assay sensitivity evaluation, female genomic DNA was serially diluted (65 ng to 0.065 pg) as templates, with ddH_2_O as a negative control.

### 2.4. Design and Selection of RAA Primer

Four pairs of RAA primers (Table 1) targeting the W-linked EE0.6 sequence (GenBank accession no. D85614.1) were designed using Primer Premier 5.0 (PREMIER Biosoft International, Palo Alto, CA, USA) based on the conserved EE0.6 gene sequence on the W chromosomes. The primer pair that specifically amplified the EE0.6 gene in females but not in males was selected for subsequent experiments. In parallel, four pairs of RAA primers (Table S1) targeting the highly conserved UCE sequence (GenBank accession no. JQ331584.1) were designed using the same criteria as an internal amplification control. All primers were synthesized by Sangon Biotech Co., Ltd. (Shanghai, China) and screened by RAA reaction using female and male genomic DNA. The EW4 amplicon was purified and directly sequenced by Sanger sequencing using the EW4 forward and reverse primers (Sangon Biotech, Shanghai, China). The obtained sequence was aligned against the reference chicken EE0.6 sequence (GenBank accession no. D85614.1) to confirm its identity.

### 2.5. Design of ssDNA, gDNA and Probe

A 60-nt single-stranded DNA (ssDNA) fragment was synthesized from the central region of the EE0.6–RAA amplification product. Five complementary guide DNAs (gDNAs) were designed at two-base intervals. All gDNAs were phosphorylated using T4 polynucleotide kinase (New England Biolabs, Ipswich, MA, USA). A probe within the primer amplification region was labeled with carboxyfluorescein (FAM) as the fluorophore and Black Hole Quencher-1 (BHQ-1) as the quencher (Table 2). For the UCE–RAA amplification product, a 60-nt ssDNA, five gDNAs (Ug-1–Ug-5), and one probe (UCE-Probe) were designed using the same strategy. The corresponding sequences are listed in Table S2.

### 2.6. Optimization of RAA Reaction Conditions

RAA was carried out using an RAA nucleic acid amplification kit (Zhongce Bio-Sci and Tech Co., Ltd., Hangzhou, China). The 50-μL reaction mixture contained one tube of lyophilized RAA powder, 29.4 μL of buffer A, 2 μL each of forward and reverse primers (10 μM), 1 μL of DNA template, 13.1 μL of ddH_2_O, and 2.5 μL of buffer B. The reaction was incubated at 40 °C for 30 min. Different temperatures (37, 38, 39, 40, 41, and 42 °C) and reaction times (10, 15, 20, 30, and 40 min) were evaluated.

### 2.7. gDNA Selection

Five candidate gDNAs were screened using a *Pf*Ago-mediated ssDNA cleavage assay. The 20-μL *Pf*Ago reaction mixture contained 2.0 μL of 10× reaction buffer (150 mM Tris–HCl, 1 M NaCl, pH 8.0), 2.66 mM *Pf*Ago (JiaoHong Biotech, Shanghai, China), 2 μL of ssDNA, 150 nM gDNA, 5 mM MgSO_4_, and 500 nM probe, with ddH_2_O to 20 μL. These conditions were used only for initial gDNA screening. Reactions were performed in a CFX Connect Real-Time PCR Detection System (Bio-Rad) at 95 °C for 30 min, with FAM fluorescence recorded every 30 s. Cleavage products were visualized using a blue-light illumination (Beijing Labgic Technology Co., Ltd., Beijing, China).

### 2.8. Establishment of RAA-PfAgo Assay

RAA was performed under optimized conditions. Subsequently, 2 μL of the RAA product was added to a 20-μL *Pf*Ago reaction mixture containing 2 μL of 10× reaction buffer (150 mM Tris–HCl, 1 M NaCl, pH 8.0), 2.66 mM *Pf*Ago, 150 nM gDNA, 5 mM MgSO_4_, and 500 nM probe. These conditions were used for the initial establishment of the RAA-*Pf*Ago assay. The reaction mixture was incubated at 95 °C for 30 min, during which the double-stranded RAA amplicon was thermally unwound into single-stranded DNA and subsequently cleaved by gDNA-guided *Pf*Ago. FAM fluorescence was recorded every 30 s. Products were visualized using a fluorescence detector or blue-light illumination (Labgic). For visual interpretation, a sample was considered positive when its endpoint fluorescence intensity was markedly higher than that of the negative control (ddH_2_O) and was clearly distinguishable by eye under blue light. Samples showing only weak or ambiguous signals that were not clearly distinguishable from the negative control were classified as negative.

### 2.9. Evaluation of the RAA-PfAgo for Sex Identification

To assess specificity, genomic DNA from six individuals of known sex (three females and three males) was tested for both the EE0.6–RAA-*Pf*Ago assay and the UCE–RAA-*Pf*Ago assay. These six individuals were from the captive population described in Section 2.1, and their sex was independently determined by conventional CHD1 PCR and adult morphology. For sensitivity, ten-fold serial dilutions of feather-derived DNA were used as templates. For the EE0.6–RAA-*Pf*Ago assay, dilutions ranged from 65 ng to 0.065 ng. For the UCE–RAA-*Pf*Ago assay, dilutions ranged from 30 ng to 0.03 pg. DdH_2_O was used as a negative control for both assays.

### 2.10. Sex Identification Using RAA-PfAgo Assay for Field Test

Feather samples from 19 Swinhoe’s pheasants of unknown sex were collected from the 25 individuals described in Section 2.1. Genomic DNA was extracted and analyzed using the EE0.6–RAA-*Pf*Ago assay, the UCE–RAA-*Pf*Ago assay, and conventional PCR. The UCE–RAA-*Pf*Ago assay was used to monitor DNA quality and amplification success, ensuring that negative EE0.6 results were not caused by failed DNA extraction or reaction inhibition. Results were compared to evaluate method performance.

### 2.11. Statistics and Analysis

All experiments were performed in at least three independent replicates. Fluorescence data were analyzed using GraphPad Prism software (version 9.5, San Diego, CA, USA). Results are presented as mean ± standard error of the mean (SEM). Statistical significance was determined using one-way analysis of variance (ANOVA) followed by Dunnett’s multiple comparisons test or two-sample *t*-test, as appropriate. A *p*-value < 0.05 was considered statistically significant.

## 3. Results

### 3.1. Overview of the RAA-PfAgo Assay Mechanism

**Figure 1:**
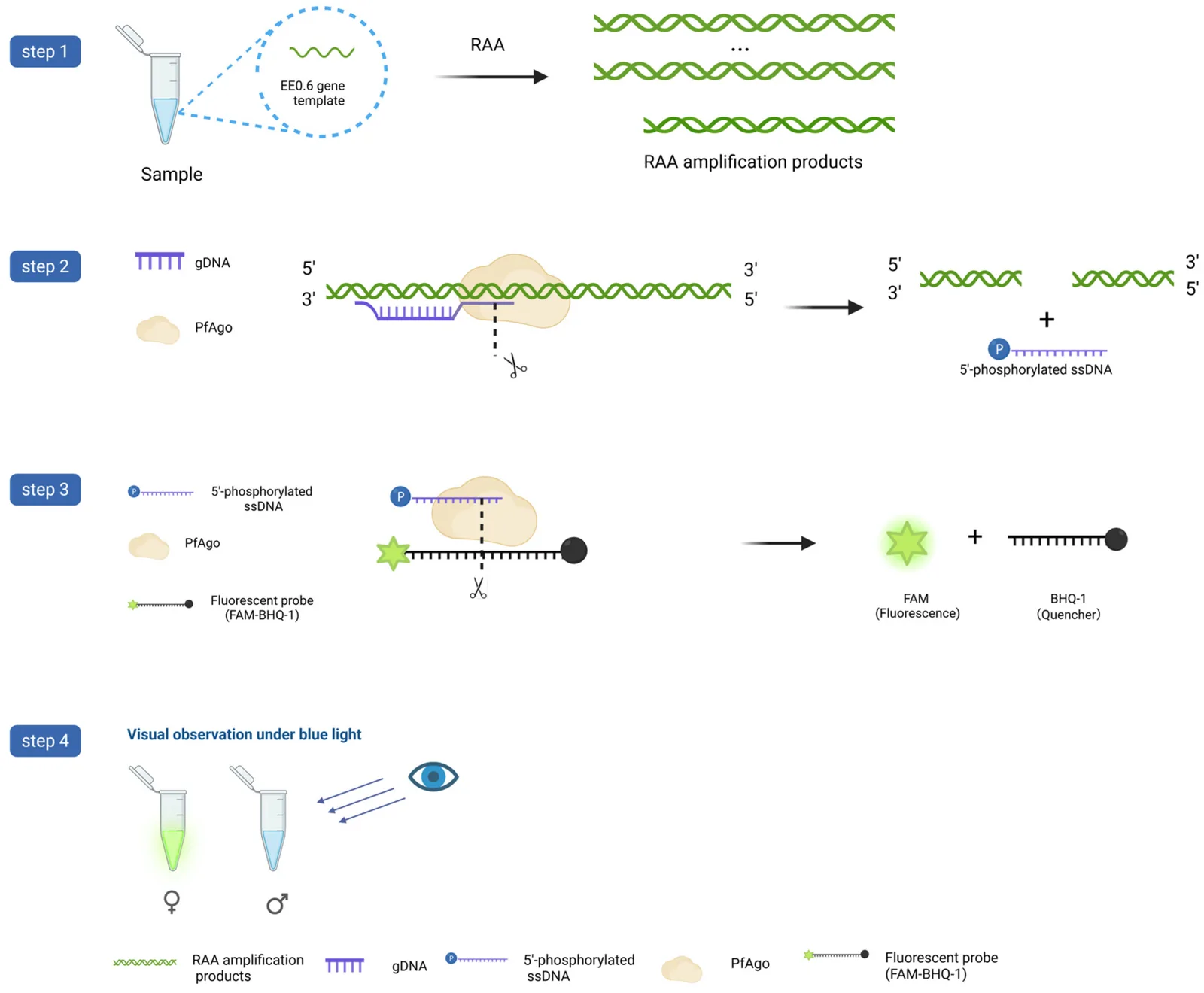
The working mechanism of the RAA-*Pf*Ago system.

The assay mechanism proceeds as follows (Figure 1): First, RAA specifically amplifies the EE0.6 gene. Then, under gDNA guidance, *Pf*Ago cleaves the RAA products. The resulting 5′-phosphorylated ssDNA acts as a new gDNA for a second cleavage round, where a fluorescent probe (FAM-BHQ1) is cleaved, releasing fluorescence detectable under blue light. Compared with CRISPR/Cas-based detection, which often requires PAM sequences and suffers from off-target collateral effects [24], the PAM-free and cascade cleavage properties of *Pf*Ago offer greater target flexibility and potential signal amplification without compromising specificity. This mechanistic advantage makes the RAA-*Pf*Ago system particularly suitable for applications where the target region lacks convenient PAM sites or where single-nucleotide resolution is required.

### 3.2. RAA Primer Selection and Optimization of Reaction Conditions

**Figure 2:**
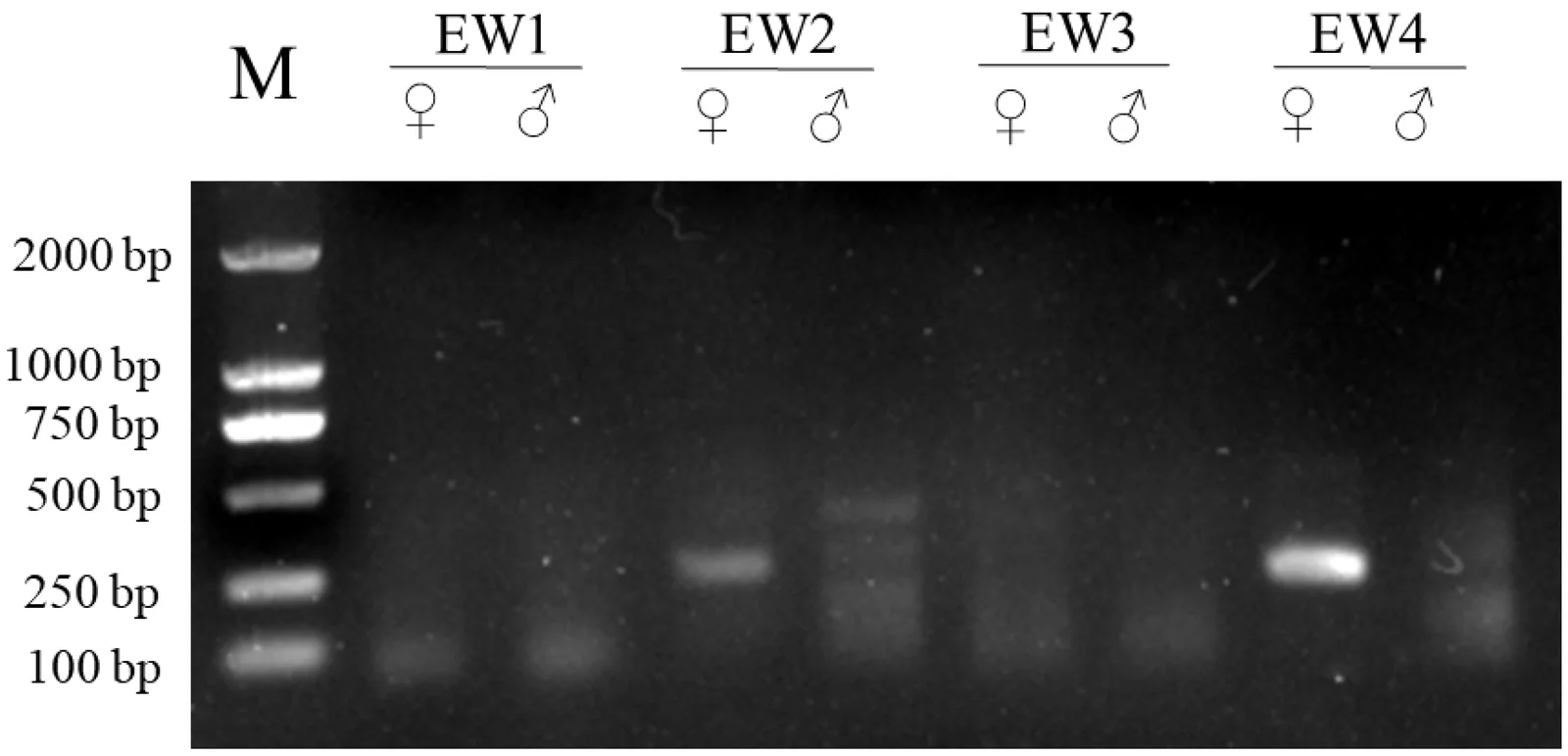
RAA primer selection for EE0.6 gene. The RAA products amplified with the basic RAA kit and four primer sets were analyzed by electrophoresis on a 1.5% agarose gel. ♀: female, ♂: male.

Genomic DNA from female and male individuals was used as templates to screen for RAA primers. After incubation at 39 °C for 30 min, the EE0.6 RAA products were analyzed by 1.5% agarose gel electrophoresis (Figure 2). Among the four primer pairs, EW4 specifically amplified the EE0.6 gene fragment and exhibited relatively high amplification intensity without multiple fragments, indicating that EW4 is the most suitable primer pair for RAA. Therefore, EW4 was selected for subsequent experiments. Sanger sequencing of the EW4 amplicon showed high sequence identity to the reference chicken EE0.6 sequence (GenBank accession no. D85614.1), confirming that the amplicon corresponds to the W-linked EE0.6 sequence (Figure S1). The UCE–RAA primers were screened under the same conditions. The UCE2 primer pair produced a bright and clear band, whereas the other primer pairs showed weaker or nonspecific amplification (Figure S2). Therefore, UCE2 was selected for the UCE-IAC.

**Figure 3:**
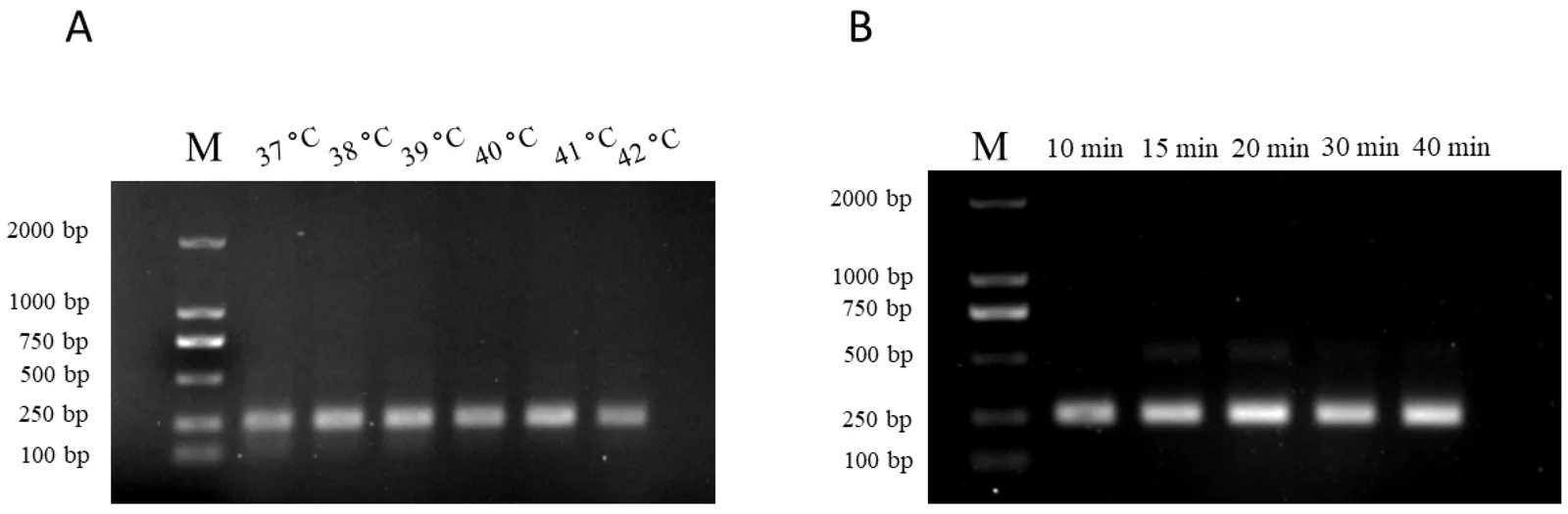
Optimization of the EE0.6-RAA assay. (**A**) Evaluation of different incubation temperatures. (**B**) Evaluation of the effect of different incubation times. RAA was amplified using the basic RAA kit and analyzed by electrophoresis on a 1.5% agarose gel.

For the EE0.6–RAA assay, as shown in Figure 3A, the target band was clearly visible in the 37–40 °C range, with maximum amplification intensity achieved at 39 °C, whereas amplification efficiency decreased markedly when the temperature exceeded 41 °C. The reaction time was also optimized (Figure 3B). Amplification products were detectable after 10 min, reached sufficient intensity at 20 min, and showed no significant improvement upon prolonged incubation. Therefore, 20 min was selected as the optimal reaction time. Considering both amplification efficiency and detection speed, the optimal conditions for EE0.6–RAA amplification were determined to be 39 °C for 20 min. The UCE–RAA assay was optimized under the same conditions. The target band intensity increased from 10 to 20 min and remained comparable from 20 to 40 min (Figure S3). Therefore, the optimal conditions for the UCE–RAA assay were also determined to be 39 °C for 20 min.

### 3.3. gDNA Selection Result

**Figure 4:**
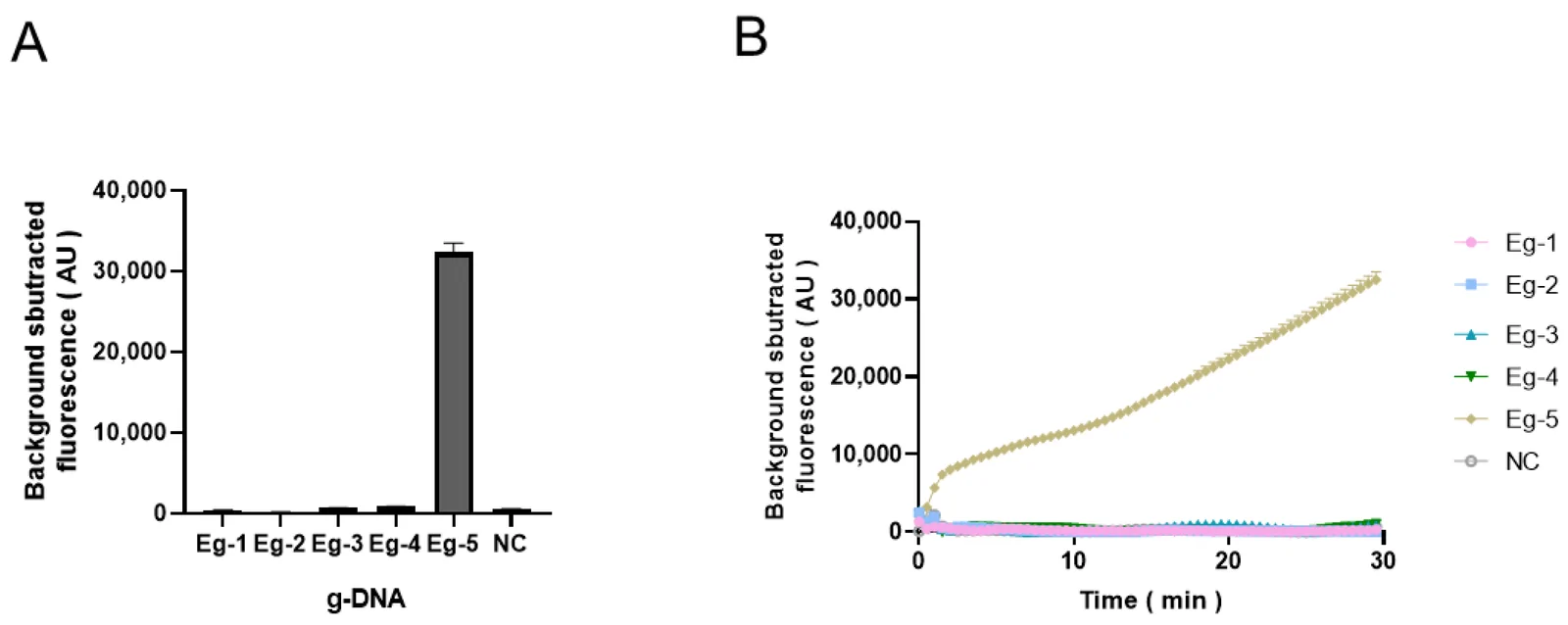
Selection of gDNA templates for the EE0.6-RAA-*Pf*Ago assay. (**A**) Endpoint fluorescence values of different gDNA templates (bar chart). (**B**) Temporal variation in fluorescence intensity of different gDNA templates (line chart).

For the EE0.6–RAA-*Pf*Ago assay, screening of these gDNAs was performed using the *Pf*Ago-mediated ssDNA cleavage assay (Figure 4A,B). Only Eg-5 produced a significantly higher endpoint fluorescence signal than the negative control (NC), while the signals from Eg-1 to Eg-4 remained at background levels (Figure 4A). Real-time fluorescence monitoring further showed that Eg-5 exhibited a rapid and continuous increase in fluorescence intensity, whereas the other candidates displayed flat curves indistinguishable from that of the NC (Figure 4B). Therefore, Eg-5 was selected as the optimal gDNA for subsequent RAA-*Pf*Ago assays. The gDNAs for the UCE–RAA-*Pf*Ago assay were screened under the same conditions. Among the five candidate gDNAs (Ug-1 to Ug-5), Ug-3 showed the highest endpoint fluorescence and the most efficient cleavage kinetics (Figure S4). Therefore, Ug-3 was selected as the optimal gDNA for the UCE–RAA-*Pf*Ago assay.

### 3.4. Optimization of RAA-PfAgo Assay

**Figure 5:**
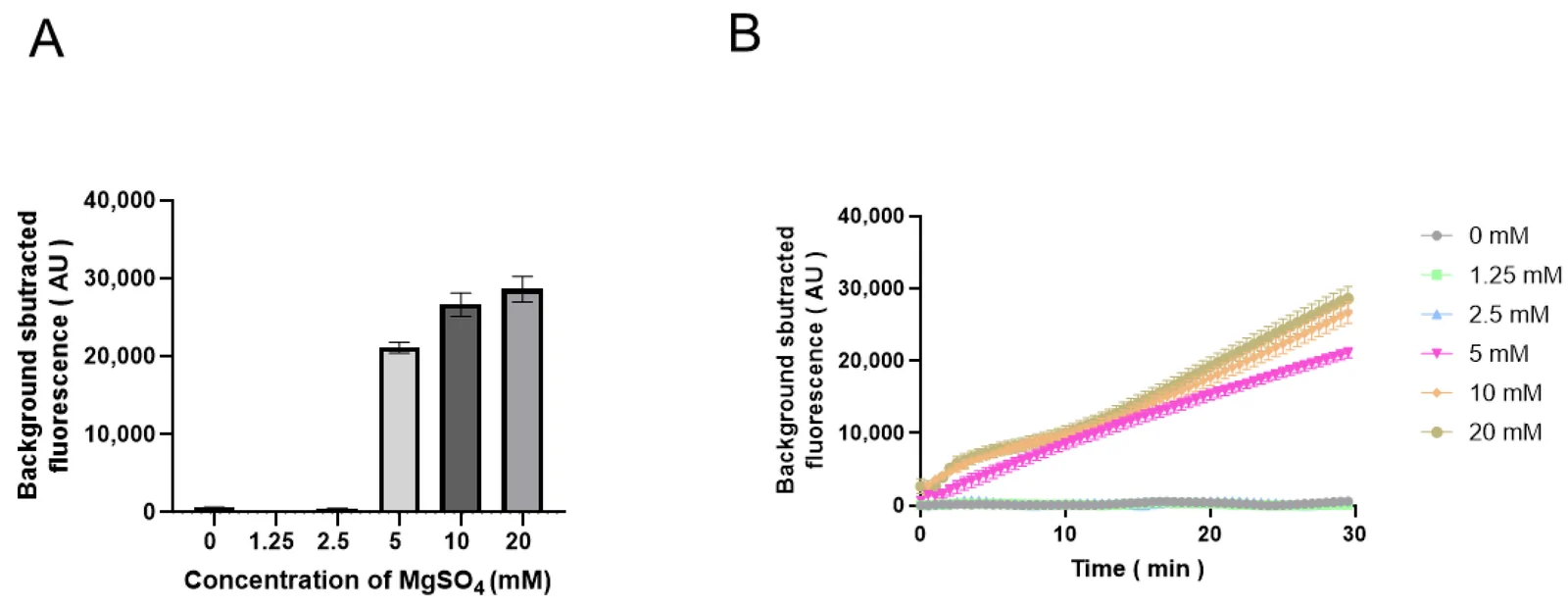
Optimization of Mg^2+^ concentration for the EE0.6-RAA-*Pf*Ago assay. (**A**) Endpoint fluorescence values at different Mg^2+^ concentrations (bar chart). (**B**) Temporal changes in fluorescence intensity at different Mg^2+^ concentrations (line chart).

*Pf*Ago activity is dependent on divalent cations. In this study, Mg^2+^ was used as the cofactor, and *Pf*Ago showed minimal cleavage activity at concentrations below 2.5 mM, with fluorescence intensity increasing markedly at 5 mM and reaching the maximum at 20 mM (Figure 5A,B). Although the 20 mM condition did not reach a clear plateau within the 30-min reaction window, it consistently produced the highest fluorescence intensity and the steepest initial reaction rate among all tested concentrations, indicating superior enzymatic activity. Thus, 20 mM MgSO_4_ was determined to be the optimal concentration within the tested range for the EE0.6–RAA-*Pf*Ago assay. However, for the UCE–RAA-*Pf*Ago assay, the highest endpoint fluorescence was observed at 10 mM Mg^2+^, with a slight decrease at 20 mM, while cleavage efficiency was comparable among 5, 10, and 20 mM (Figure S5). Therefore, 10 mM MgSO_4_ was selected to be the optimal concentration within the tested range for the UCE–RAA-*Pf*Ago assay.

**Figure 6:**
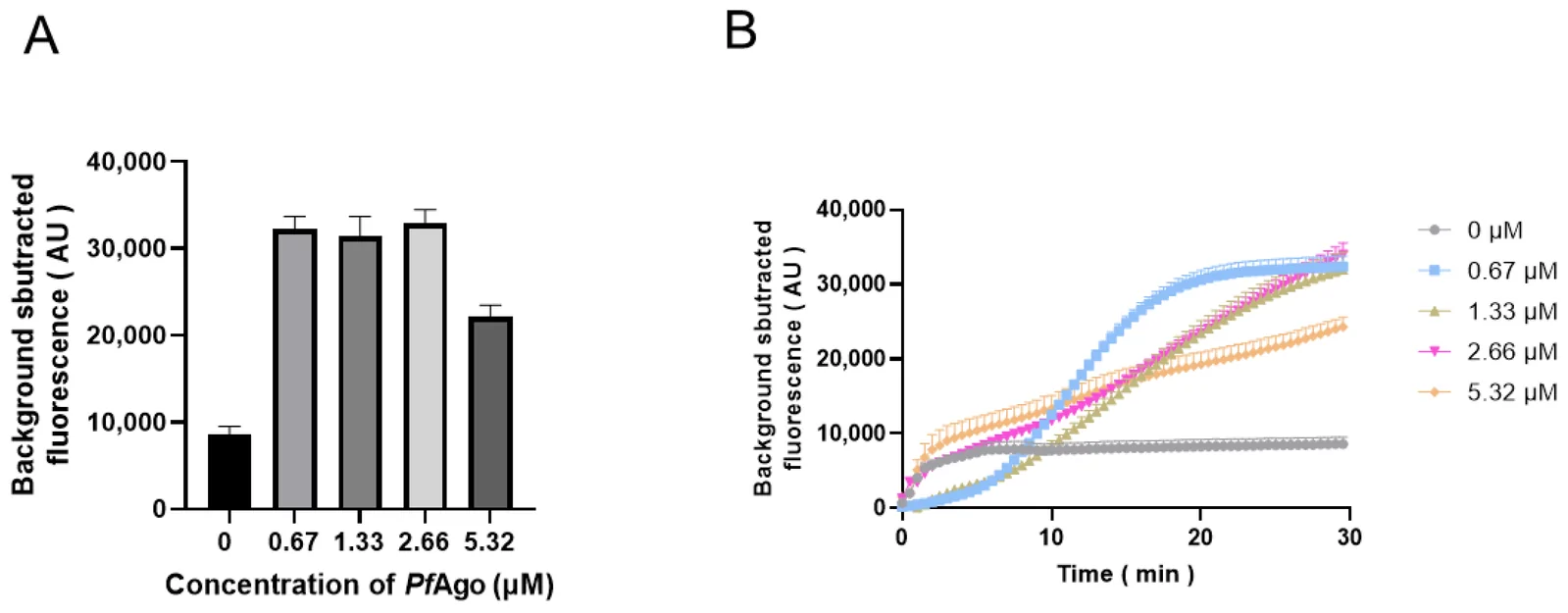
Optimization of *Pf*Ago Concentration for the EE0.6-RAA-*Pf*Ago assay. (**A**) Endpoint fluorescence values at different *Pf*Ago concentrations (bar chart). (**B**) Temporal changes in fluorescence intensity at different *Pf*Ago concentrations (line chart).

The optimal *Pf*Ago concentration was then investigated by testing 0.67, 1.33, 2.66, and 5.32 μM for the EE0.6–RAA-*Pf*Ago assay. Negligible fluorescence was observed without *Pf*Ago, while the signal increased with enzyme concentration. The strongest and fastest fluorescence response was obtained at 0.67 μM, with higher concentrations yielding no further improvement and even a slight decrease (Figure 6A,B). All reactions were completed within 30 min. At 0.67 μM, the fluorescence signal reached the plateau faster than at higher concentrations, indicating efficient cleavage with minimal enzyme usage. Therefore, 0.67 μM was selected as the optimal *Pf*Ago concentration. For the UCE–RAA-*Pf*Ago assay, both endpoint fluorescence and cleavage efficiency increased from 0.67 to 2.66 μM and then decreased at 5.32 μM, with the highest signal at 2.66 μM (Figure S6). Therefore, 2.66 μM was selected as the optimal *Pf*Ago concentration for the UCE–RAA-*Pf*Ago assay.

**Figure 7:**
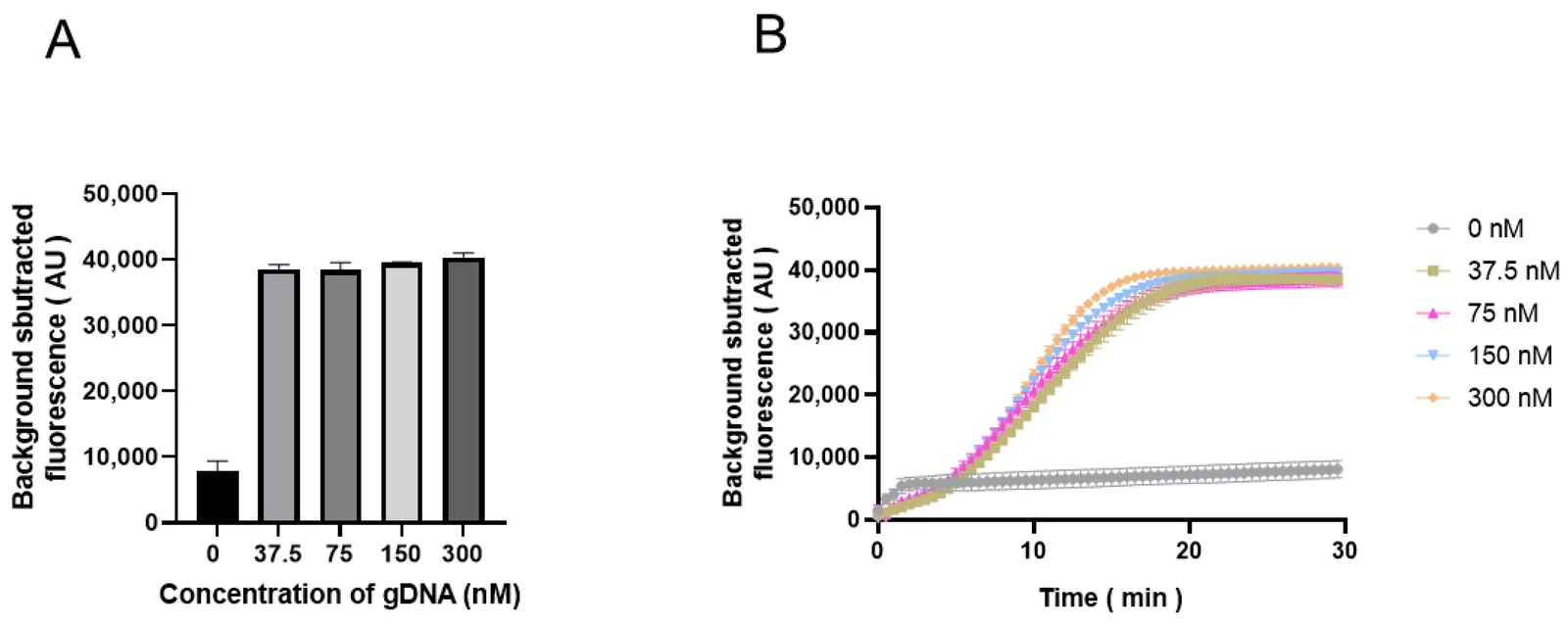
Optimization of gDNA concentration for the EE0.6-RAA-*Pf*Ago assay. (**A**) Endpoint fluorescence values at different gDNA concentrations (bar chart). (**B**) Temporal changes in fluorescence intensity at different gDNA concentrations (line chart).

Finally, gDNA concentrations ranging from 37.5 to 300 nM were optimized for the EE0.6–RAA-*Pf*Ago assay. Endpoint fluorescence analysis revealed the highest signal intensity at 300 nM (Figure 7A), which was further confirmed by real-time kinetics showing the fastest reaction rate and earliest plateau (Figure 7B). Among the tested concentrations, 300 nM gDNA yielded the highest signal intensity and was therefore selected for subsequent experiments. For the UCE–RAA-*Pf*Ago assay, both endpoint fluorescence and cleavage efficiency increased continuously from 37.5 to 300 nM, with the highest signal at 300 nM (Figure S7). Therefore, 300 nM gDNA was also selected as the optimal concentration within the tested range for the subsequent UCE–RAA-*Pf*Ago assay.

Based on these optimization experiments, the final conditions for the EE0.6–RAA-*Pf*Ago assay were determined to be 0.67 μM *Pf*Ago, 300 nM gDNA, 20 mM MgSO_4_, and 500 nM probe. For the UCE–RAA-*Pf*Ago assay, the final conditions were 2.66 μM *Pf*Ago, 300 nM gDNA, 10 mM MgSO_4_, and 500 nM probe. These optimized conditions were used in all subsequent experiments.

### 3.5. Specificity and Sensitivity of RAA-PfAgo Assay

**Figure 8:**
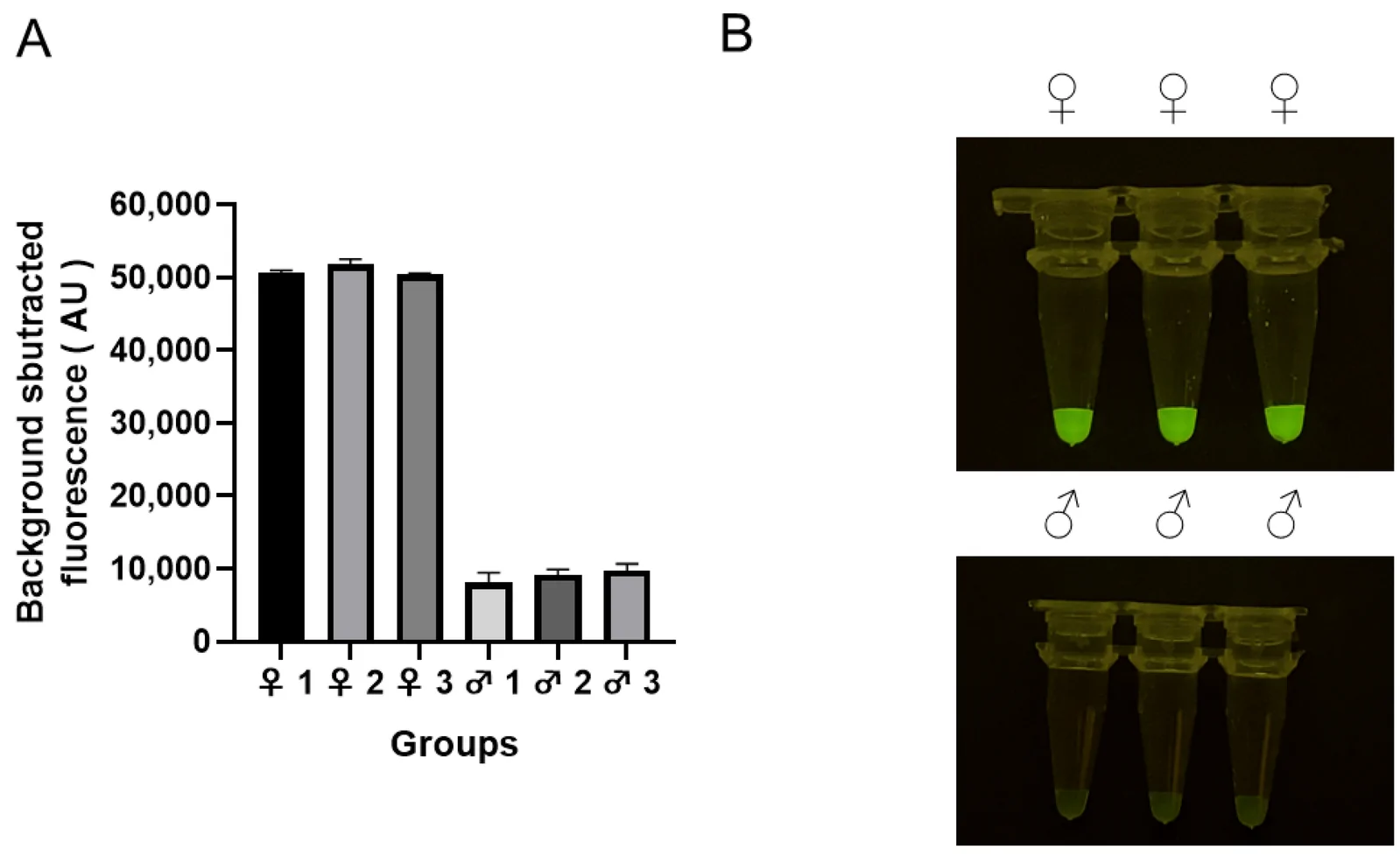
Specificity evaluation of the EE0.6-RAA-*Pf*Ago assay for female and male samples. (**A**) Endpoint fluorescence values of RAA-PfAgo for different samples. (**B**) Observations under blue light. ♀: female, ♂: male.

The specificity of the RAA-*Pf*Ago system was evaluated using six genomic DNA samples of known sex (three females and three males) from Swinhoe’s pheasant. As shown in Figure 8A, fluorescence signals were detected exclusively in female samples, with no signal observed in males, demonstrating a clear sex-dependent response for the EE0.6–RAA-*Pf*Ago assays. Visual inspection under blue light (Figure 8B) yielded consistent results, confirming the high specificity of the assay for female sex identification. For the UCE–RAA-*Pf*Ago assays, both female and male samples produced positive signals (Figure S8), confirming that the internal control amplifies in both sexes.

**Figure 9:**
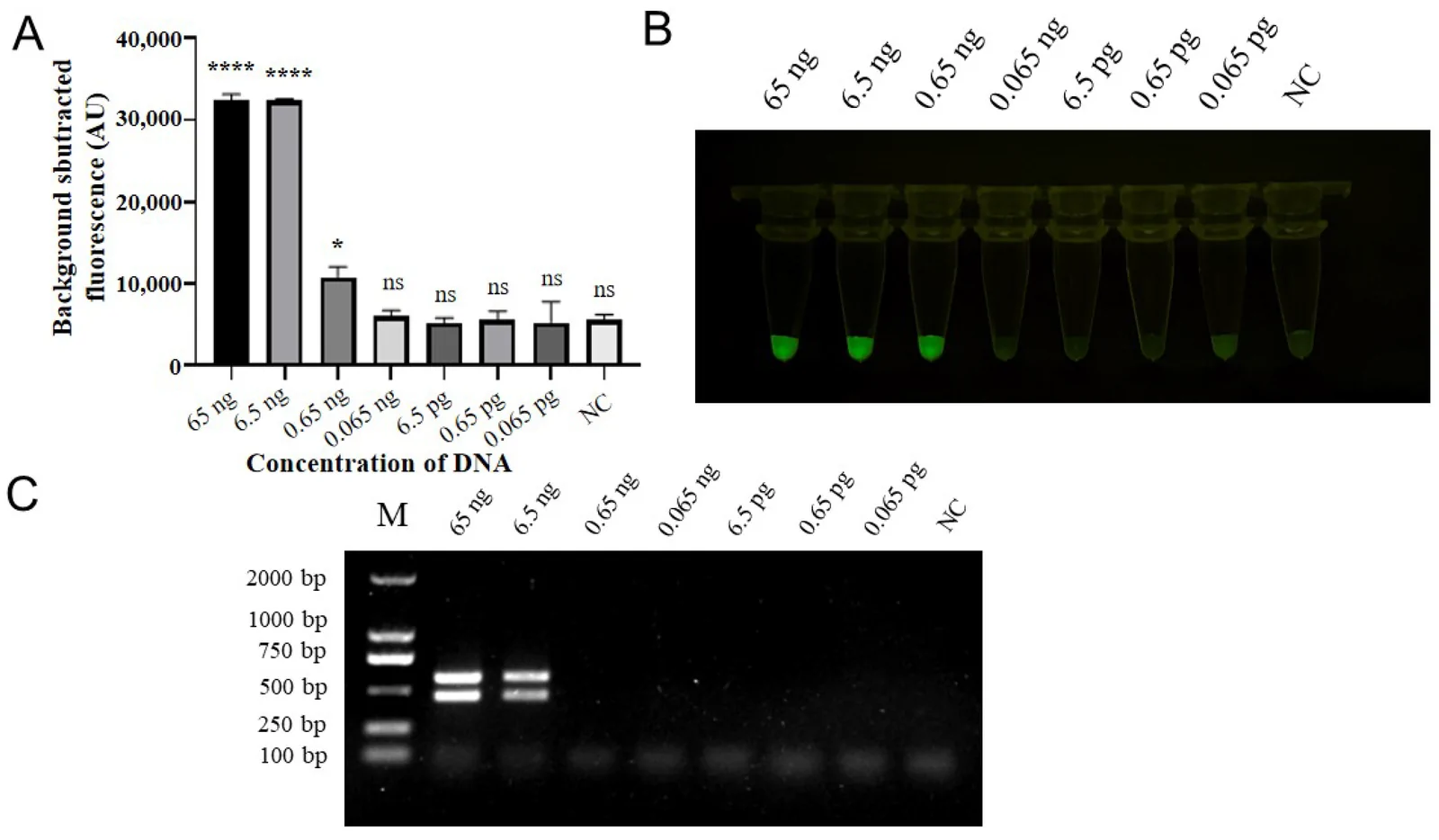
Sensitivity validation of the EE0.6-RAA-*Pf*Ago and PCR assays. (**A**) Endpoint fluorescence analysis of RAA-*Pf*Ago sensitivity. (**B**) Visual detection of RAA-*Pf*Ago products under blue light. (**C**) Sensitivity of the PCR assay evaluated by 1.5% agarose gel electrophoresis. NC, negative control. Error bars represent SEM (*n* = 3). * *p* < 0.05, **** *p* < 0.0001, ns not significant.

To determine the sensitivity of the EE0.6–RAA-*Pf*Ago assays, ten-fold serial dilutions of female genomic DNA were tested, ranging from 65 ng to 0.065 pg. The results for each dilution were as follows: 65 ng, 6.5 ng, and 0.65 ng were positive, whereas 0.065 ng, 6.5 pg, 0.65 pg, and 0.065 pg were negative. No fluorescence signal was detected at 0.065 ng or below. Therefore, the detection limit of the EE0.6–RAA-*Pf*Ago assay for Swinhoe’s pheasant was determined to be 0.65 ng. In contrast, the detection limit of conventional PCR was 6.5 ng (Figure 9C). For the UCE–RAA-*Pf*Ago assay, positive signals were observed at 30, 3, and 0.3 ng, whereas no signal was detected below 0.3 ng (Figure S9). Therefore, the detection limit of the UCE–RAA-*Pf*Ago internal control was 0.3 ng.

### 3.6. Field Sample Test of RAA-PfAgo Assay

**Figure 10:**
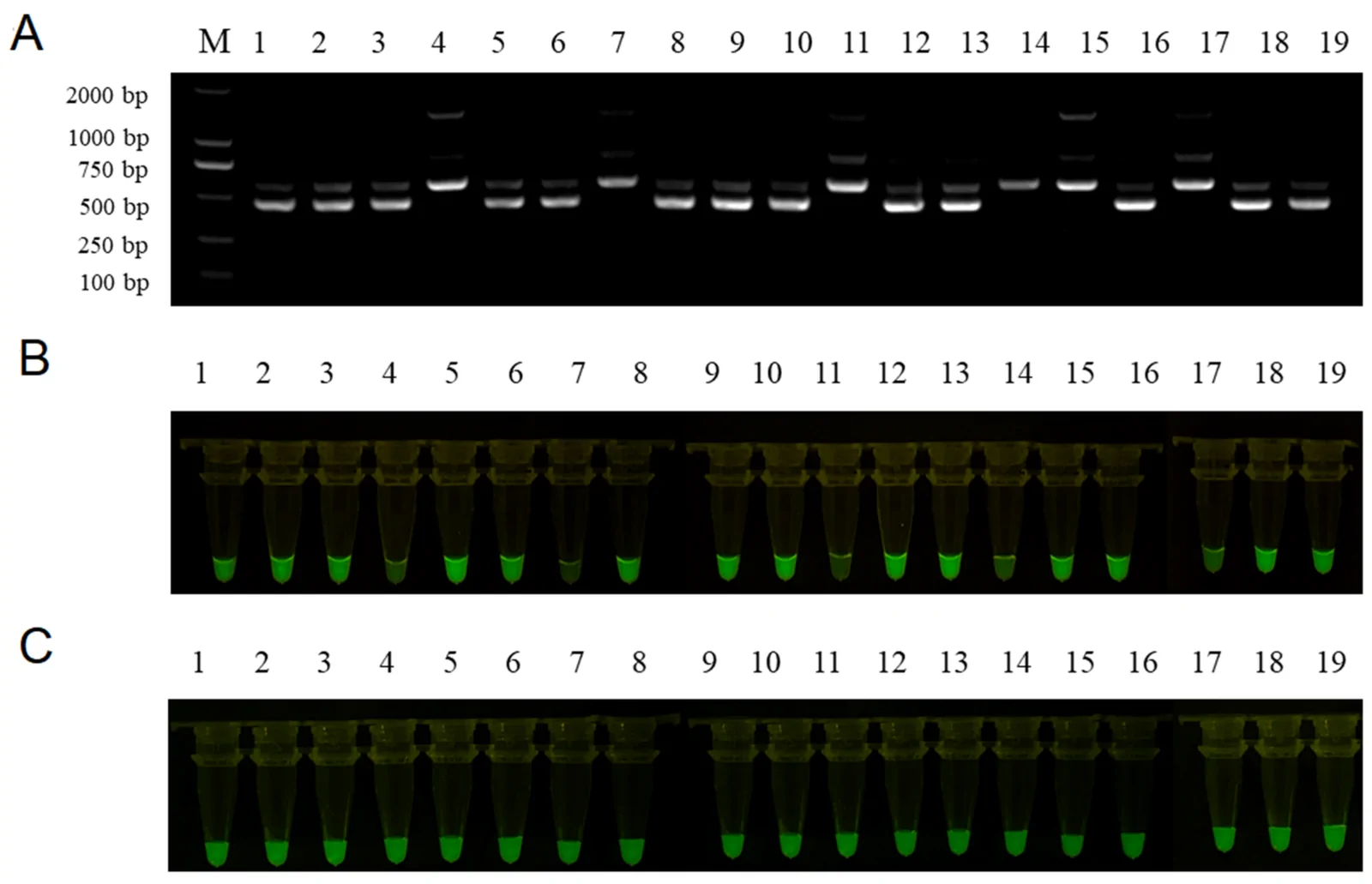
Field tests of conventional PCR and RAA-*Pf*Ago assays. (**A**) Detection of field samples by conventional PCR, analyzed by 1.5% agarose gel electrophoresis. (**B**) Detection of the same field samples by EE0.6-RAA-*Pf*Ago assay, visualized under blue light. (**C**) Detection of the same clinical samples by UCE-RAA-*Pf*Ago assay, visualized under blue light.

To verify the reliability of the EE0.6–RAA-*Pf*Ago method for sex determination in juvenile Swinhoe’s pheasant, 19 genomic DNA samples from individuals of unknown sex were analyzed in parallel using both the established RAA-*Pf*Ago method and conventional PCR. The EE0.6–RAA-*Pf*Ago assay identified samples 1, 2, 3, 5, 6, 8, 9, 10, 12, 13, 16, 18, and 19 as female, whereas samples 4, 7, 11, 14, 15, and 17 showed no fluorescence signal and were classified as male (Figure 10B). These results were in complete agreement with those obtained from conventional PCR (Figure 10A). For the UCE–RAA-*Pf*Ago assay, all 19 samples produced clear positive signals (Figure 10C), confirming successful DNA extraction and amplification. This indicates that the RAA-*Pf*Ago method is a reliable and accurate tool for sex determination in Swinhoe’s pheasant.

## 4. Discussion

Accurate sex determination is critical for optimizing population structure and breeding efficiency in captive management and reintroduction programs [8,9]. The EE0.6–RAA-*Pf*Ago assay developed in this study demonstrated analytical specificity and sensitivity for sex identification in Swinhoe’s pheasant. In our dilution experiments, the detection limit was 10-fold lower than that of conventional PCR. This improvement is particularly significant for non-invasive samples (e.g., feathers) where DNA yield is often low and degraded, as it reduces the risk of false negatives due to insufficient template [21,25].

To further ensure the reliability of negative results, a UCE-IAC was established and validated using the same RAA-*Pf*Ago workflow. The UCE–RAA-*Pf*Ago assay targets a highly conserved avian sequence and amplifies in both females and males. Its detection limit was 0.3 ng, and all 19 clinical samples produced positive UCE–RAA-*Pf*Ago assay signals, confirming successful DNA extraction and amplification. Therefore, the negative EE0.6 results in male samples can be confidently interpreted as true negatives rather than false negatives caused by DNA degradation or reaction inhibition. This internal control provides an additional layer of quality assurance for the RAA-*Pf*Ago assay.

With this quality-control assurance in place, we further compared the RAA-*Pf*Ago assay with other molecular sexing methods across multiple operational dimensions. Conventional PCR, while being the most validated method for avian sexing, requires thermal cycling (2–3 h runtime), gel electrophoresis, and a detection limit of 6.5 ng DNA in this study, rendering it impractical for field conservation work. Among isothermal techniques, LAMP offers rapid amplification but suffers from complex primer design (six primers), high susceptibility to non-specific amplification, and reliance on turbidity or colorimetric indicators that lack sequence specificity [26]. The recently developed RAA/RPA-CRISPR/Cas systems (e.g., Cas12a) share similarities with our approach but strictly require PAM sequences for target recognition [27,28]. In our EE0.6 amplicon, the absence of convenient PAM sites within the optimal primer-binding region would have precluded CRISPR-based detection. Moreover, CRISPR/Cas systems exhibit collateral trans-cleavage activity, which, while useful for signal amplification, can cause off-target effects and complicate quantitative interpretation. In contrast, our RAA-*Pf*Ago system offers unique advantages: (i) it is PAM-free, providing greater target flexibility; (ii) its cascade cleavage mechanism provides intrinsic signal amplification without collateral activity, ensuring cleaner signal generation [20]; (iii) the blue-light visual readout eliminates the need for any fluorescence reader or electrophoresis apparatus; and (iv) it achieves a detection limit of 0.65 ng, which is 10-fold more sensitive than conventional PCR and superior or comparable to other isothermal methods. These features collectively position the RAA-*Pf*Ago method as a practical, cost-effective, and robust solution for field-based sex identification in endangered monomorphic bird species.

In this study, four candidate RAA primer pairs targeting the EE0.6 gene were evaluated. The EE0.6 sequence has been reported to be widely conserved on the avian W chromosome across a broad range of Carinatae birds [12,29]. In this study, it was used as the target for sex identification in Swinhoe’s pheasant. Among the four primer pairs, EW4 exhibited the highest amplification efficiency and specificity. The superior performance of EW4 is likely attributed to its localization within regions that are well conserved in EE0.6 and absent from the Z chromosome, allowing specific amplification from DNA with interernce from male DNA [30]. The other three primer pairs (EW1–EW3) either produced non-specific bands or exhibited weaker amplification, possibly because their binding sites are less accessible or less conserved with the EE0.6 sequence, leading to inefficient amplification or non-specific products. The EW4 primers were designed based on the conserved EE0.6 sequence from the chicken (GenBank accession no. D85614.1) and are therefore not strictly specific to Swinhoe’s pheasant. Since EE0.6 is highly conserved across Carinatae birds. However, cross-species amplification was not experimentally tested in this study. Due to the limited availability of EE0.6 sequences from closely related species in public databases, a comprehensive cross-species alignment was not included. The results regarding the optimization of reaction temperature indicate that efficient amplification occurs only within a narrow range of 37–40 °C, reaching peak performance at 39 °C. Such reactions are highly sensitive to temperature fluctuations [31]; at lower temperatures, reduced enzyme activity may hinder the formation of recombinase-primer complexes, thereby slowing down the kinetics of the amplification reaction [32]. Conversely, when the temperature exceeds 41 °C, amplification efficiency declines significantly; this is likely attributable to compromised stability of the recombinase-DNA complexes and an increase in non-specific interactions [33]. The optimal reaction temperature identified in this study is highly consistent with previously reported RAA and RPA systems, which typically operate within the 37–42 °C range [34]. The optimized 39 °C amplification condition enables the assay to operate using low-power portable heaters, highlighting its practical applicability for field-based sex identification in conservation programs. This temperature sensitivity underscores the importance of precise temperature control in field settings, although portable battery-powered heaters are now capable of maintaining such ranges with ±1 °C accuracy. Furthermore, similar temperature-dependent performance characteristics were observed in RAA-*Pf*Ago and RPA-*Pf*Ago systems that incorporate the *Pf*Ago protein [35]. This suggests that maintaining the optimal amplification temperature is not only critical for the upstream amplification reaction but is equally indispensable for ensuring the efficient execution of the downstream *Pf*Ago-mediated signal generation process. Upstream amplification efficiency directly determines downstream cleavage efficiency. It should be noted that the 95 °C incubation step serves a dual purpose in the *Pf*Ago detection. It thermally unwinds the double-stranded RAA amplicon into single-stranded DNA, and it simultaneously provides the optimal catalytic temperature for *Pf*Ago, which exhibits robust cleavage activity at 87–99 °C [36]. This allows the gDNA guides to bind the target strand as it unwinds and enables efficient *Pf*Ago-mediated cleavage.

The choice of gDNA (Eg-5) was critical for cleavage efficiency, as binding site accessibility and secondary structure can vary. Notably, the failure of Eg-1 to Eg-4 may be attributed to either the inaccessibility of their target sequences within the ssDNA secondary structure or suboptimal alignment between the gDNA and the cleavage site, which is known to affect *Pf*Ago’s cleavage efficiency [37,38]. Mg^2+^ was selected as the cofactor for its compatibility with isothermal amplification [39,40]; cleavage activity increased with Mg^2+^ concentration, consistent with previous reports [41]. However, high Mg^2+^ concentrations may promote non-specific cleavage in some Argonaute systems [42]; in this study, no significant non-specific cleavage was observed above the background threshold defined in Methods 2.8, possibly because the reaction time was limited to 30 min and the probe design was highly specific. Field validation using 19 feather samples showed 100% concordance with PCR, confirming reliability. The use of non-invasive feather samples is particularly valuable for endangered species, as it eliminates the need for blood collection, which can cause stress and injury in vulnerable or protected bird populations.

Limitations include the need for further validation in complex field samples (e.g., environmental feces or degraded museum specimens), the potential for nonspecific amplification under suboptimal conditions, and the current two-step procedure (RAA followed by *Pf*Ago detection), which increases operational complexity and the risk of carryover contamination. Although DNA extraction currently requires a basic laboratory, future integration with instrument-free extraction methods such as alkaline lysis could further simplify the workflow and make the assay more suitable for field deployment. Moreover, the specificity evaluation was based on six individuals with known sex (three females and three males). While the 19 clinical samples showed 100% concordance with conventional PCR, their sexes were initially unknown; therefore, a larger set of known-sex individuals (at least 20–30) will be needed in future studies to fully assess primer binding site polymorphisms and further validate the assay. In addition, future studies should include blinded replicate samples, including weak/borderline signals, and report agreement statistics such as Cohen’s kappa to further establish the reproducibility of the visual classification. Cross-species validation in other avian species was not performed in this study and warrants further investigation. In addition, while the assay successfully discriminated females from males, it does not provide information on Z-linked markers that could be useful for identifying sex-reversed individuals or confirming ZZ/ZW karyotypes in cases of abnormal development. Future studies should explore the lyophilization of all reagents into a single tube to enable one-step, cold-chain-free delivery [17]. Future efforts should focus on a one-tube RAA-*Pf*Ago system and validation with larger and more diverse sample sets.

## 5. Conclusions

In summary, we successfully developed a rapid, sensitive, and visually interpretable RAA-*Pf*Ago assay targeting the W-linked EE0.6 sequence for sex identification in Swinhoe’s pheasant. The assay achieved 10-fold higher sensitivity (0.65 ng) than conventional PCR (6.5 ng) and reduced turnaround time by approximately one hour. Operating at constant temperature without a thermal cycler and enabling visual readout under blue light, this method provides a robust, field-deployable solution for early-stage sex management in Swinhoe’s pheasant and potentially other endangered monomorphic bird species.

## Figures and Tables

**Table 1:** Sequences of the primers targeting the EE0.6 gene for the RAA assay in this study.

Primers	Sequence (5′-3′)	Size (bp)	Tm (°C)	GC (%)
EW1	F: ACTTGAGATAGTGACTTCCTTCTGGCAAAG	426	64.7	43.3
	R: CCATCTTCTAAGTAATGGGTTCAGGTTGAG		63.3	43.3
EW2	F: TAGCAGTTATGGTCCTATGCCTACCACATT	261	65.3	43.3
	R: TTGTTGGCACTGATTTAGTTCCTTGCTTGT		65.8	40.0
EW3	F: AGGGAGTATCTAGCAGTTATGGTCCTATGC	384	65.0	46.7
	R: CCATCTTCTAAGTAATGGGTTCAGGTTGAG		63.3	43.3
EW4	F: TTATGGTCCTATGCCTACCACATTCCTATT	257	63.4	40.0
	R: TGTTGTTGGCACTGATTTAGTTCCTTGCTT		65.8	40.0

**Table 2:** ssDNA, gDNA, and probe sequences targeting the EE0.6 gene used in this study.

Name	Sequence (5′-3′)
ssDNA	AAATACTAAGCATAAGCAATCATGTTAACATTAGGGTCACTGAATTTTACTTAAAAGTTT
Eg-1	TTTTAAGTAAAATTCA
Eg-2	TTAAGTAAAATTCAGT
Eg-3	AAGTAAAATTCAGTGA
Eg-4	GTAAAATTCAGTGACC
Eg-5	AAAATTCAGTGACCCT
EE-Probe	FAM-AACTTTTAAGTAAAATTCAGTGAC-BHQ1

## Data Availability

The original contributions presented in this study are included in the article/Supplementary Material. Further inquiries can be directed to the corresponding author(s).

## Supplementary Materials

The following supporting information can be downloaded at https://www.mdpi.com/article/10.3390/cimb48101002/s1.
