# Supplementary material for: Development of a Rapid, Sensitive, and Visual Recombinase-Aided Amplification Combined with Pyrococcus furiosus Argonaute (RAA-PfAgo) Assay for Sex Identification in Swinhoe’s Pheasant (Lophura swinhoii)
Source: Curr Issues Mol Biol. 2026 Sep 29;48(10):1002. doi: 10.3390/cimb48101002 (PMC13703586; doi:10.3390/cimb48101002)
Supplement: Supplementary file 1 [file cimb-48-01002-s001.zip › Figure S1-S9.pdf]

a

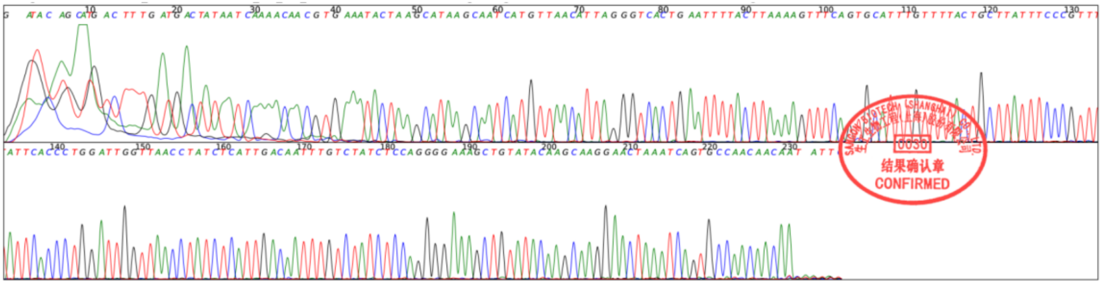

b

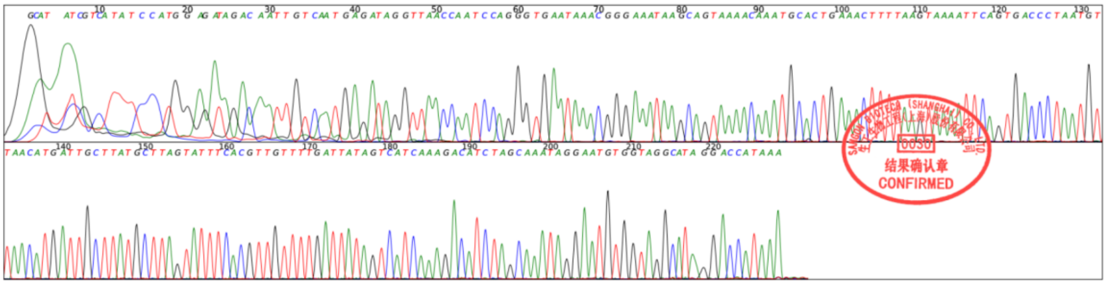

c

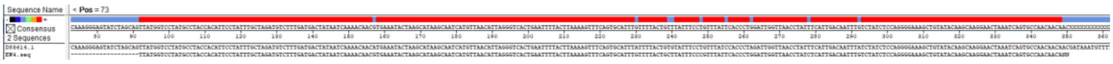

Figure S1. Sanger sequencing confirmation of the EW4-RAA amplicon. (**a**) The purified EW4-RAA amplicon was directly sequenced using the EW4 forward primers. (**b**) The purified EW4-RAA amplicon was directly sequenced using the EW4 reverse primers. (**c**) The alignment against the reference chicken EE0.6 sequence (GenBank accession no. D85614.1) confirms that the amplicon corresponds to the W-linked EE0.6 sequence.

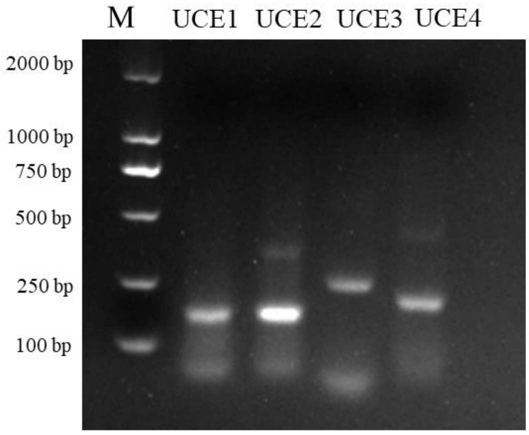

Figure S2. RAA primer selection for UCE gene. The RAA products amplified with the basic RAA kit and four primer sets were analyzed by electrophoresis on a 1.5% agarose gel.

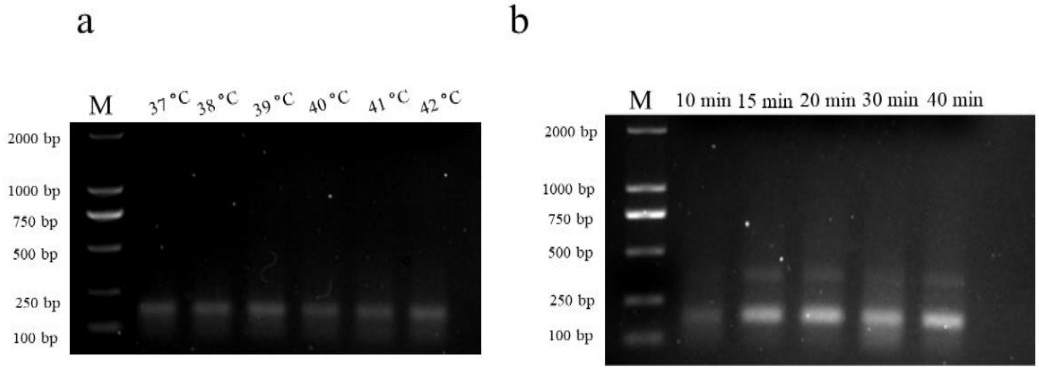

Figure S3. Optimization of the UCE-RAA assay. (**a**) Evaluation of different incubation temperatures. (**b**) Evaluation of the effect of different incubation times. RAA amplified using the basic RAA kit and analyzed by electrophoresis on a 1.5% agarose gel.

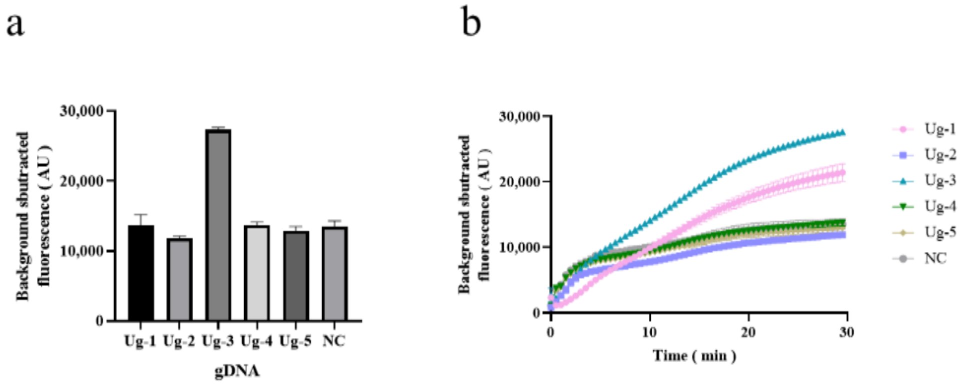

Figure S4. Selection of gDNA templates for the UCE-RAA-*Pf*Ago assay. (**a**) Endpoint fluorescence values of different gDNA templates (bar chart). (**b**) Temporal variation in fluorescence intensity of different gDNA templates (line chart).

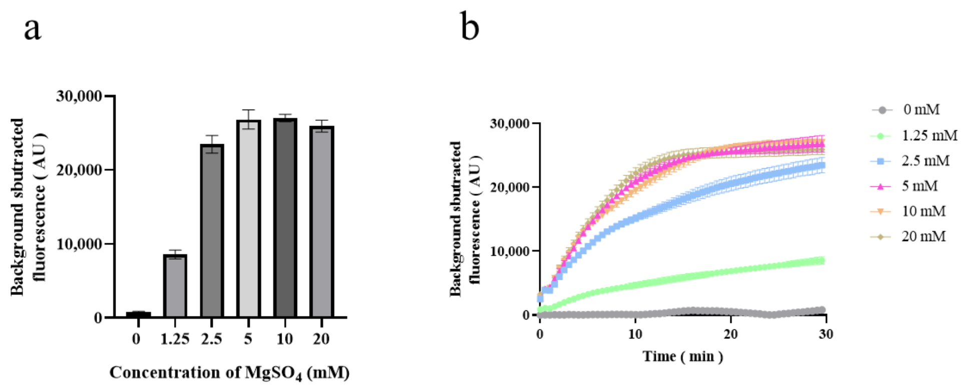

Figure S5. Optimization of Mg^{2+} concentration for the UCE-RAA-*Pf*Ago assay. (**a**) Endpoint fluorescence values at different Mg^{2+} concentrations (bar chart). (**b**) Temporal changes in fluorescence intensity at different Mg^{2+} concentrations (line chart).

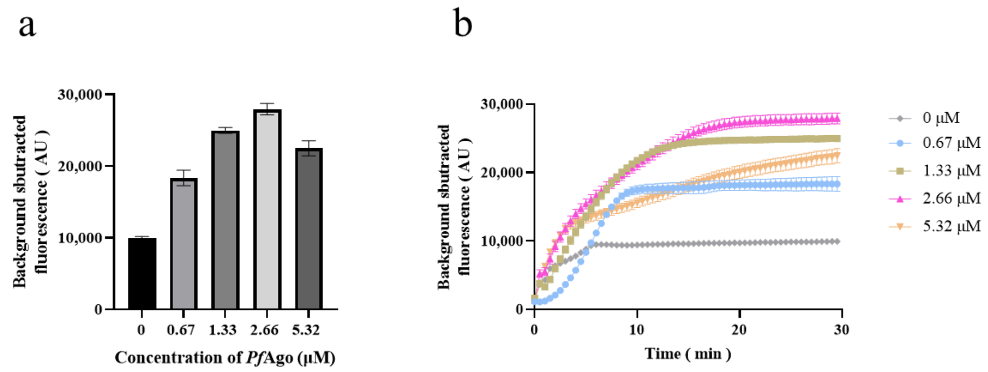

Figure S6. Optimization of *Pf*Ago Concentration for the UCE-RAA-*Pf*Ago assay. (**a**) Endpoint fluorescence values at different *Pf*Ago concentrations (bar chart). (**b**) Temporal changes in fluorescence intensity at different *Pf*Ago concentrations (line chart).

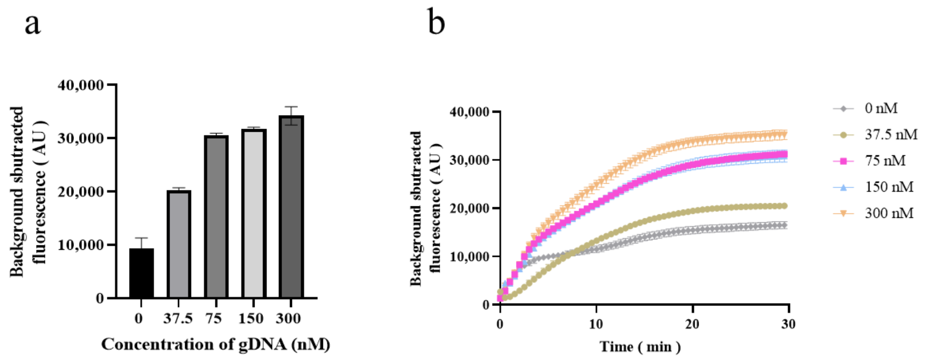

Figure S7. Optimization of gDNA concentration for the UCE-RAA-*Pf*Ago assay. (a) Endpoint fluorescence values at different gDNA concentrations (bar chart). (**b**) Temporal changes in fluorescence intensity at different gDNA concentrations (line chart).

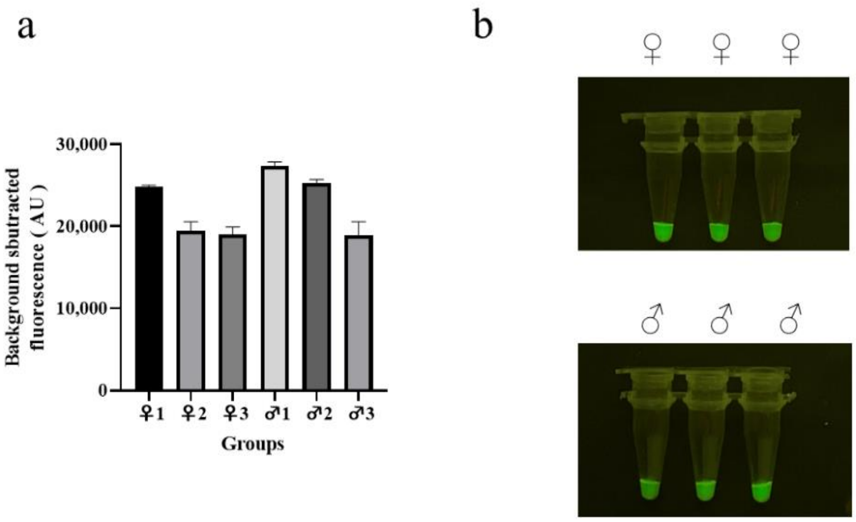

Figure S8. Specificity evaluation of the UC-RAA-*Pf*Ago assay for female and male samples. (**a**) Endpoint fluorescence values of RAA-*Pf*Ago for different samples. (**b**) Observations under blue light. ♀: female, ♂: male.

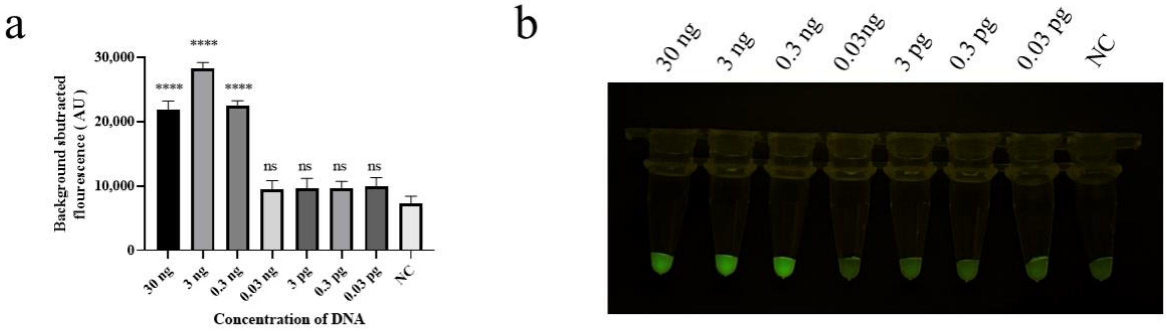

Figure S9. Sensitivity validation of the UCE-RAA-*Pf*Ago and PCR assays. (**a**) Endpoint fluorescence analysis of RAA-*Pf*Ago sensitivity. (**b**) Visual detection of RAA-*Pf*Ago products under blue light. NC, negative control. Error bars represent SEM (n = 3). *$P < 0.05$, ****$P < 0.0001$, ns not significant.
