# Supplementary material for: Development of a Rapid, Sensitive, and Visual Recombinase-Aided Amplification Combined with Pyrococcus furiosus Argonaute (RAA-PfAgo) Assay for Sex Identification in Swinhoe’s Pheasant (Lophura swinhoii)
Source: Curr Issues Mol Biol. 2026 Sep 29;48(10):1002. doi: 10.3390/cimb48101002 (PMC13703586; doi:10.3390/cimb48101002)
Supplement: Supplementary file 1 [file cimb-48-01002-s001.zip › Table S1-S2.pdf]

Table S1. Sequences of the primers targeting the UCE gene for the RAA assay in this study.

Primers	Sequence (5′-3′)	Size (bp)	Tm (°C)	GC (%)
UCE1	F: CTTTAGTTAAGAAATTCGCTCGCCATTATG	174	65.9	36.7
	R: CATTTTAATACCCTGCAGAACAGTCCTCAT		67.9	40.0
UCE2	F: TCTTTGGTCTTTAGTTAAGAAATTCGCTCG	174	66.6	36.7
	R: TACCCTGCAGAACAGTCCTCATAACTCATC		69.9	46.7
UCE3	F: CTTTAGTTAAGAAATTCGCTCGCCATTATG	252	65.9	36.7
	R: TTTATTGCCCAGAAAATTCCATTCTGCTAT		66.8	33.3
UCE4	F: ATTATGGCATCATTAGGGAAACAAGGATAA	208	66.0	33.3
	R: ATTCTGCTATCTGATTCGAAAAGTCCAGTC		67.6	40.0

Table S2. ssDNA, gDNA, and probe sequences targeting the UCE gene used in this study.

Name	Sequence (5′-3′)
ssDNA	CATTATGGCATCATTAGGGAAACAAGGATAAAATTACTCCATGGTTATCCTT GTTTCCCTA
Ug-1	TTTATCCTTGTTTCCC
Ug-2	ATTTTATCCTTGTTTC
Ug-3	ATTTTATCCTTGTTTC
Ug-4	TAATTTTATCCTTGTT
Ug-5	AGTAATTTTATCCTTG
UCE-Probe	FAM-TGGAGTAATTTTATCCTTGTTTCCCTAA-BHQ1
